# Supplementary material for: Optimizing Xenium In Situ data utility by quality assessment and best-practice analysis workflows
Source: Nat Methods. 2025 Mar 13;22(4):813–23. doi: 10.1038/s41592-025-02617-2 (PMC11978515; doi:10.1038/s41592-025-02617-2)
Supplement: Supplementary file 1 — Supplementary Table 1 legend. [file 41592_2025_2617_MOESM1_ESM.pdf]

nature methods

Supplementary information https://doi.org/10.1038/s41592-025-02617-2

Optimizing Xenium In Situ data utility by quality assessment and best-practice analysis workflows

In the format provided by the authors and unedited

Supplementary Information

Supplementary Table 1- Main characteristics Xenium datasets

Supplementary Table 1 provides key metrics for each of the 25 Xenium datasets, including cellular density, total read counts, gene and read distribution per cell, and spatial area. Metrics such as the proportion of reads assigned to cells, mean reads and genes per cell, and quality measures (e.g., reads with QV >20) offer a concise view of the dataset quality and coverage across samples, supporting cross-dataset comparisons.
