# Supplementary material for: Optimizing Xenium In Situ data utility by quality assessment and best-practice analysis workflows
Source: Nat Methods. 2025 Mar 13;22(4):813–23. doi: 10.1038/s41592-025-02617-2 (PMC11978515; doi:10.1038/s41592-025-02617-2)
Supplement: Supplementary file 2 — Reporting Summary [file 41592_2025_2617_MOESM2_ESM.pdf]

nature portfolio

Corresponding author(s): Marco Salas, Sergio Nilsson, Mats

Last updated by author(s): 19012024

Reporting Summary

Nature Portfolio wishes to improve the reproducibility of the work that we publish. This form provides structure for consistency and transparency in reporting. For further information on Nature Portfolio policies, see our Editorial Policies and the Editorial Policy Checklist.

Statistics

For all statistical analyses, confirm that the following items are present in the figure legend, table legend, main text, or Methods section.

n/a	Confirmed	
☐	☒	The exact sample size (*n*) for each experimental group/condition, given as a discrete number and unit of measurement
☐	☒	A statement on whether measurements were taken from distinct samples or whether the same sample was measured repeatedly
☒	☐	The statistical test(s) used AND whether they are one- or two-sided<br>*Only common tests should be described solely by name; describe more complex techniques in the Methods section.*
☐	☒	A description of all covariates tested
☐	☒	A description of any assumptions or corrections, such as tests of normality and adjustment for multiple comparisons
☐	☒	A full description of the statistical parameters including central tendency (e.g. means) or other basic estimates (e.g. regression coefficient) AND variation (e.g. standard deviation) or associated estimates of uncertainty (e.g. confidence intervals)
☒	☐	For null hypothesis testing, the test statistic (e.g. *F*, *t*, *r*) with confidence intervals, effect sizes, degrees of freedom and *P* value noted<br>*Give P values as exact values whenever suitable.*
☒	☐	For Bayesian analysis, information on the choice of priors and Markov chain Monte Carlo settings
☐	☒	For hierarchical and complex designs, identification of the appropriate level for tests and full reporting of outcomes
☐	☒	Estimates of effect sizes (e.g. Cohen's *d*, Pearson's *r*), indicating how they were calculated

Our web collection on statistics for biologists contains articles on many of the points above.

Software and code

Policy information about availability of computer code

Data collection

Xenium datasets were either made available by 10X Genomics or collected using 10X Xenium instruments. Datasets were preprocessed using the code included in https://github.com/Moldia/Xenium_benchmarking v1.2.0

Data analysis

All the code used in this study can be found at https://github.com/Moldia/Xenium_benchmarking v1.2.0

Python packages used through the study include:
affine==2.4.0, anndata==0.8.0, alphashape==1.3.1, biopython==1.81, click==8.1.5, click-log==0.4.0, click-plugins==1.1.1, cloudpickle==2.1.0, contextily==1.3.0, cython==3.0.2, dask==2022.2.0, dask-image==2021.12.0, descartes==1.1.0, doubletdetection==4.2, fiona==1.9.5, geographiclib==2.0, geopandas==0.10.2, geopy==2.4.0, gprofiler-official==1.0.0, h5py==3.7.0, holoviews==1.16.2, igraph==0.9.11, imagecodecs==2021.11.20, imageio==2.21.0, leidenalg==0.8.10, libpysal==4.7.0, louvain==0.7.2, matplotlib==3.5.2, matplotlib-scalebar==0.8.1, matplotlib-venn==0.11.9, mygene==3.2.2, naivede==1.2.0, networkx==2.6.3, numba==0.56.0, numpy==1.21.6, omnipath==1.0.5, pandas==1.3.5, phenograph==1.5.7, pooch==1.7.0, pydantic==1.9.1, pynndescent==0.5.7, pyparsing==3.0.9, pyproj==3.2.1, rasterio==1.2.10, rtree==1.0.1, scanpy==1.9.1, scikit-image==0.19.3, scikit-learn==1.0.2, scipy==1.7.3, seaborn==0.11.2, shapely==2.0.1, spatialde==1.1.3, spatialdm==0.1.0, squidpy==1.2.2, statsmodels==0.13.2, tifffile==2021.11.2, toolz==0.12.0, tqdm==4.64.0, umap-learn==0.5.3, xarray==0.20.2, zarr==2.12.0

Furthermore, R packages include:
Seurat(4.3.0), SeuratObject(4.1.3), Giotto (1.1.2)

For manuscripts utilizing custom algorithms or software that are central to the research but not yet described in published literature, software must be made available to editors and reviewers. We strongly encourage code deposition in a community repository (e.g. GitHub). See the Nature Portfolio guidelines for submitting code & software for further information.

Data

Policy information about availability of data

All manuscripts must include a data availability statement. This statement should provide the following information, where applicable:

- Accession codes, unique identifiers, or web links for publicly available datasets
- A description of any restrictions on data availability
- For clinical datasets or third party data, please ensure that the statement adheres to our policy

Three types of Xenium datasets were used through the manuscript, including (1) datasets provided by 10X Genomics, (2) datasets published elsewhere and (3) datasets generated specifically for this project. First, for the 10X Genomics datasets, the original datasets used in this study can be obtained from in https://www.10xgenomics.com/datasets [03.05.2024]. In addition, the spinal cord datasets used were originally published by Kukanja & Mattsson-Langseth et al. (24). Lastly, freshly generated datasets include four mouse brain sections, labelled as "hm" through the study. Their original data can be downloaded from: https://doi.org/10.5281/zenodo.10566172.
In addition, we have also made Xenium datasets available as AnnData objects. These files can be downloaded from various¬¬ Zenodo repositories (https://doi.org/10.5281/zenodo.11124988 , https://doi.org/10.5281/zenodo.11121221 , https://doi.org/10.5281/zenodo.11120307)
All datasets used in the comparison between SRT platforms (Figure 2) are publicly available datasets. In the case of the commercial platforms , the datasets are available in the company's data portals (MERSCOPE: https://vizgen.com/data-release-program/, CosMx: https://nanostring.com/products/cosmx-spatial-molecular-imager/ffpe-dataset/, Molecular Cartography: https://resolvebiosciences.com/datasets/). For both MERFISH and HS-ISS, datasets were made available in their original publications (3,4). In addition, resegmented and regionally annotated datasets, ready to reproduce the comparison, can be found at https://doi.org/10.5281/zenodo.11619309.
Allen brain atlas single cell dataset ABC atlas) used through the study is available at https://portal.brain-map.org/atlases-and-data/bkp/abc-atlas.

Human research participants

Policy information about studies involving human research participants and Sex and Gender in Research.

Reporting on sex and gender	N/A
Population characteristics	N/A
Recruitment	N/A
Ethics oversight	N/A

Note that full information on the approval of the study protocol must also be provided in the manuscript.

Field-specific reporting

Please select the one below that is the best fit for your research. If you are not sure, read the appropriate sections before making your selection.

☒ Life sciences ☐ Behavioural & social sciences ☐ Ecological, evolutionary & environmental sciences

For a reference copy of the document with all sections, see nature.com/documents/nr-reporting-summary-flat.pdf

Life sciences study design

All studies must disclose on these points even when the disclosure is negative.

Sample size	All publicly and internally available Xenium datasets at the time of the study (25 different Xenium datasets) were used with the aim of generalize the conclusions of the study. Due to the recent commercialization of the Xenium product, limited datasets are available
Data exclusions	Cells that did not pass several quality filters based on the number of transcripts and genes detected were excluded from analysis
Replication	Due to the recent commercialization of the Xenium product, limited datasets were available through the study. As a consequence, no biological replicates could be used. Mouse brain datasets, however, presented technical replicates,which were used through the study to assess the robustness of the method.
Randomization	This study presents reduce sample size with n=1 in many cases. Thus, randomization is not relevant to this study
Blinding	Due to the limited sample size, with n=1 in most cases, blinding does not apply to this study.

Reporting for specific materials, systems and methods

We require information from authors about some types of materials, experimental systems and methods used in many studies. Here, indicate whether each material, system or method listed is relevant to your study. If you are not sure if a list item applies to your research, read the appropriate section before selecting a response.

Materials & experimental systems

n/a	Involved in the study
☒	☐ Antibodies
☒	☐ Eukaryotic cell lines
☒	☐ Palaeontology and archaeology
☒	☐ Animals and other organisms
☒	☐ Clinical data
☒	☐ Dual use research of concern

Methods

n/a	Involved in the study
☒	☐ ChIP-seq
☒	☐ Flow cytometry
☒	☐ MRI-based neuroimaging
